# Hyperkyphosis among the Elderly in a Community: A Descriptive Cross-sectional Study

**DOI:** 10.31729/jnma.7351

**Published:** 2022-08-31

**Authors:** Inosha Bimali, Sikha Pudasaini

**Affiliations:** 1 Department of Physiotherapy, Kathmandu University School of Medical Sciences, Dhulikhel, Kavre, Nepal; 2 Department of Physiotherapy, Norvic International Hospital, Thapathali, Kathmandu, Nepal

**Keywords:** *aging*, *hyperkyphosis*, *posture*

## Abstract

**Introduction:**

Aging brings progressive changes in the physiology of the musculoskeletal system that leads to postural changes and degenerative diseases in elderly. The most common postural change is hyperkyphosis which decreases physical performance, ability to perform daily activities, overall quality of life, and increases the risk of falls in elderly. The aim of the study was to find out the prevalence of hyperkyphosis among the elderly in a community.

**Methods:**

A descriptive cross-sectional study was done in a local community from 26 May 2019 to 8 June 2019 after obtaining ethical approval from the Institutional Review Committee (Protocol approval number: 138/19). Participants above 60 years of age were included in the study. Convenience sampling was done. A bubble inclinometer was used to measure the degree of kyphosis. Point estimate and 95% Confidence Interval were calculated.

**Results:**

Among 144 elderly participants, hyperkyphosis was present in 90 (62.50%) (54.59-70.41, 95% Confidence Interval) with the mean hyperkyphosis being 47.07±4.83°. The elderly in the age group of 70-75 years had a higher degree of hyperkyphosis with a mean value of 47.77±4.92°. The mean hyperkyphosis was 48.18±5.30° and 45.31±3.36° in female and male participants respectively.

**Conclusions:**

The prevalence of hyperkyphosis was found to be higher in our study compared to other studies conducted in similar settings. Early identification and interventions of hyperkyphosis are thus warranted to prevent the detrimental consequences in the later stages of life.

## INTRODUCTION

Aging is associated with progressive changes in the physiology of the musculoskeletal systems like excessive bone destruction, vertebral fractures, muscle weakness, and degenerative diseases leading to thoracic hyperkyphosis.^1^ Hyperkyphosis refers to an abnormally accentuated concavity of the thoracic spine (>40 degrees)^2^ and its prevalence in elderly are 20-40% worldwide.^3^

Hyperkyphosis impairs mobility, increases the risk of falls and fractures, and reduces the quality of life in older people.^4^ Several health conditions are associated with hyperkyphosis like impairment of lung function, decreased functional capacity, and increase mortality.^5^ With the significant negative consequences of hyperkyphosis, early identification and intervention could have important clinical and public health benefits.

The aim of the study was to find out the prevalence of hyperkyphosis among the elderly in a community.

## METHODS

A descriptive cross-sectional study was conducted among the elderly from the community of Dhulikhel from 26 May 2019 to 8 June 2019. Ethical approval was obtained from the Institutional Review Committee (Protocol approval number: 138/19) of the Kathmandu University School of Medical Sciences. Permission was also taken from the Dhulikhel municipality. Participants above 60 years who were permanent residents of Dhulikhel were included in the study whereas the elderly with structural spinal problems, inflammatory or osteo-metabolic diseases, congenital disorders of the spine, history of vertebral fractures, and surgical spine fixation or lower limb surgery were excluded from the study. Convenience sampling was done and the sample size was calculated using the following formula:


$$\begin{array}{ll} \text{n} & ={\text{Z}}^{2}\times \frac{\text{p}\times \text{q}}{{\text{e}}^{\text{2}}}\\ & =1.96^{2}\times \frac{0.316\times 0.684}{0.08^{2}}\\ & =130 \end{array}$$

Where,

n= minimum required sample size
Z= 1.96 at 95% Confidence Interval (CI)
p= prevalence of hyperkyphosis among the elderly, 31.6%^3^
q= 1-p
e= margin of error, 8%

Hence, a minimum required sample size of 130 was obtained. However, a sample size of 144 was taken in the study. The demographic data of the participants were recorded and the participants were screened according to the selection criteria. The assessment was conducted using a Bubble inclinometer which is one of the reliable and valid tools to measure hyperkyphosis.^6-8^ Prior to measuring, the bubble inclinometer was placed at a zero level to ensure an accurate starting point. The participants were asked to relax and stand comfortably, exposing their backs. The bony prominence of the C7 and T1 vertebra was identified and the inclinometer was placed between these bony prominences and the angle obtained was noted. Then, the bony prominences of T12 and L1 were identified and an inclinometer was placed between two bony prominences, and the angle was noted. The angle measured by bubble inclinometer (both at C7-T1 and T12-L1 level) was recorded to identify the kyphosis angle. An angle of more than 40 degrees was noted as hyperkyphosis.^2^

The collected data were arranged, entered, and analysed in IBM SPSS Statistics 21.0. Point estimate and 95% CI were calculated.

## RESULTS

Among 144 elderly participants, the prevalence of hyperkyphosis was 90 (62.50%) (54.59-70.41, 95% CI). The participants had a mean age of 67.13±5.26 years. The mean hyperkyphosis among them was found to be 47.07±4.83°. Among them, 76 (52.80%) were female and 68 (47.20%) were male participants. The female participants demonstrated higher hyperkyphosis than male participants. The mean hyperkyphosis was higher in age group of 71-75 years and in the C7-T1 level.

**Table 1:** Hyperkyphosis angle at different spinal levels, age category and gender (n= 90).

Parameters	Mean±Standard Deviation
**Age (in years)**	
60-65	46.14±4.67
66-70	47.45±4.92
71-75	47.77±4.92
Spinal levels^*^	
C7-T1	36.04±6.24
T12-L1	11.01±5.08
**Gender**	
Male	45.31±3.36
Female	48.18±5.30

* C7-7^th^ cervical vertebra, T1-1^st^ thoracic vertebra, T12-12^th^ thoracic vertebra, L1-1^st^ lumbar vertebra

## DISCUSSION

Aging and postural abnormalities have always been a concern for elderly. In this study, the prevalence of hyperkyphosis was 62.5% and the mean hyperkyphosis was 47.07±4.83°. Few literature have reported the prevalence and incidence of hyperkyphosis in elderly to vary between 20% to 40% among both men and women.^5,9^ Our study showed a higher prevalence and degree of hyperkyphosis in both genders compared to the findings from the previous literature. However, a similar study done in China also reported the prevalence of hyperkyphosis among Chinese elderly to be higher 75.2% with a mean hyperkyphosis of 49.0°.^10^ A similar degree of mean score of hyperkyphosis has also been reported in several studies conducted in the United States.^11,12^ The cause for the increase in kyphosis could be due to multiple reasons like degeneration of the intervertebral disc,^13^ vertebral wedging and compression fractures,^14^ spinal extensor muscle weakness,^15^ decreased spinal extension mobility,^16^ calcification, and ossification of anterior longitudinal ligament of the thoracic region,^17^ short pectoral and hip muscles,^18^ and age-related somatosensory, visual and vestibular deficits contributing to the loss of upright postural control.^19^

Tall stature is considered one of the factors for increased hyperkyphosis and it is positively associated with spinal deformities.^20^ Nepalese despite having short stature compared to the western population have shown an increased degree of hyperkyphosis.^21^ Height could be a disputable factor for bringing an actual change in spinal deformities. A study done in Japan reported that increased kyphosis was found in the community who were mostly involved in agriculture and farming due to the sustained posture attained by the farmers while performing their work.^9^ As reported by the International labour organization, 65% of Nepalese are involved in agriculture^22^ and this could also be one of the reasons for the increased kyphosis among Nepalese elderly.

Another significant finding of the study was an increased kyphosis angle at the C7-T1 vertebral level than at the T12-L1 vertebral level. Literature has reported the C5-C6 and C6-C7 to be the most degenerated vertebral level in the participants with thoracic and lumbar deformities.^23,24^ This could be due to the positional changes in older people like increased downward gazing or forward gazing during daily activities which increases the loading in the noncontractile structure and stretches the anterior neck structures. This position further shortens the posterior muscles leading to increased muscular tension increasing the pressure in the cervical disc and leading to early degeneration^25^ which could precipitate hyperkyphosis in older people.

The study also found female participants to have greater thoracic kyphosis than males. Studies have shown that kyphosis angle rapidly increases after 40 years in females.^26^ One of the causes could be postmenopausal hormonal changes which cause weakening of spinal ligaments that lead to increased osteoporotic changes.^27^ Prevalence of osteoporosis was found to be significantly more in the middle-aged women of Nepal than the women from western countries and the reasons could be due to low dietary intake, low socioeconomic status, frequent childbirth, and low educational level.^28^ A study done in China reported that there is an increased incidence of osteoporosis in the Asian population which could be due to the high prevalence of Vitamin D insufficiency.^29^ Early osteoporotic changes in the Nepalese population could be one of the factors causing postural deviations contributing to hyperkyphosis in elderly.

Literature has reported that an excessive degree of hyperkyphosis could increase the risk of falls and trauma affecting the overall quality of life of older people. Since the mean kyphotic angle of Nepalese older people is found to be higher compared to the other countries, a future study is thus recommended on identifying the incidence of risk of falls due to hyperkyphosis in older Nepalese adults.

There were a few limitations in the study. This was a descriptive cross-sectional study so an association between the study variables could not be made. Also, the limited sample size and the single-centric nature of the study limit its generalizability of the study.

## CONCLUSIONS

The prevalence of hyperkyphosis and the mean hyperkyphosis were found to be higher in community-dwelling Nepalese elderly compared to similar studies conducted in similar settings in other countries. Early identification and interventions of hyperkyphosis could be helpful to prevent detrimental consequences in the later stages of life.
